# RETRACTED: Thomas et al. Actively Targeted Nanodelivery of Echinomycin Induces Autophagy-Mediated Death in Chemoresistant Pancreatic Cancer In Vivo. *Cancers* 2020, *12*, 2279

**DOI:** 10.3390/cancers17243888

**Published:** 2025-12-05

**Authors:** Alexandra Thomas, Abhilash Samykutty, Jorge G. Gomez-Gutierrez, Wenyuan Yin, Michael E. Egger, Molly McNally, Phillip Chuong, William M. MacCuaig, Sabrin Albeituni, Matthew Zeiderman, Min Li, Barish H. Edil, William E. Grizzle, Kelly M. McMasters, Lacey R. McNally

**Affiliations:** 1 Department of Hematology Oncology, Wake Forest Baptist Health, Winston-Salem, NC 27157, USA; althomas@wakehealth.edu; 2 Stephenson Cancer Center, University of Oklahoma, Oklahoma City, OK 73104, USA; abhilash9samykutty21@gmail.com (A.S.); mwmcnally@hotmail.com (M.M.); Bmaccuaig9@gmail.com (W.M.M.); 3 Department of Surgery, University of Louisville, Louisville, KY 40202, USA; jorge.gutierrez@louisville.edu (J.G.G.-G.); Wenyuan.Yin@osumc.edu (W.Y.); michael.egger@louisville.edu (M.E.E.); phillip.chuong@louisville.edu (P.C.); kelly.mcmasters@louisville.edu (K.M.M.); 4 Department of Cancer Predisposition, St. Jude Children’s Research Hospital, Memphis, TN 38105, USA; Sabrin.Albeituni@STJUDE.ORG; 5 Department of Surgery, University of California Davis, Sacramento, CA 95616, USA; mrzeiderman@ucdavis.edu; 6 Department of Surgery, University of Oklahoma, Oklahoma City, OK 73104, USA; Min-li@ouhsc.edu (M.L.); Barish-Edil@ouhsc.edu (B.H.E.); 7 Department of Pathology, University of Alabama Birmingham, Birmingham, AL 35294, USA; wgrizzle2@gmail.com

The journal retracts the article, “Actively Targeted Nanodelivery of Echinomycin Induces Autophagy-Mediated Death in Chemoresistant Pancreatic Cancer In Vivo” [1], cited above.

Following publication, concerns were brought to the attention of the Editorial Office regarding multiple irregularities found in images presented in this article [1].

Adhering to our standard procedure, an investigation was conducted by the Editorial Office and Editorial Board, which confirmed indications of inappropriate image editing contained within Figures 3A and 6C. While the authors fully collaborated with this process, they were unable to provide supporting material for the Editorial Board evaluation. As a result, the Editorial Board has lost confidence in the reliability of the findings and decided to retract this publication [1], as per MDPI’s retraction policy (https://www.mdpi.com/ethics#_bookmark30).

This retraction was approved by the Editor-in-Chief of the journal *Cancers* journal.

The authors did not agree to this retraction.
